# Malnutrition and infant and young child feeding in informal settlements in Mumbai, India: findings from a census

**DOI:** 10.1002/fsn3.214

**Published:** 2015-03-03

**Authors:** Abigail Bentley, Sushmita Das, Glyn Alcock, Neena Shah More, Shanti Pantvaidya, David Osrin

**Affiliations:** 1 UCL Institute for Global Health, Institute of Child Health30 Guilford Street, London, WC1N 1EH, UK; 2 SNEHA (Society for Nutrition, Education and Health Action), Urban Health Centre, Chota Sion Hospital60 Feet Road, Shahunagar, Dharavi, Mumbai, 400017, Maharashtra, India

**Keywords:** Anthropometry, child, preschool, India, malnutrition, Mumbai, poverty areas

## Abstract

Childhood malnutrition remains common in India. We visited families in 40 urban informal settlement areas in Mumbai to document stunting, wasting, and overweight in children under five, and to examine infant and young child feeding (IYCF) in children under 2 years. We administered questions on eight core WHO IYCF indicators and on sugary and savory snack foods, and measured weight and height of children under five. Stunting was seen in 45% of 7450 children, rising from 15% in the first year to 56% in the fifth. About 16% of children were wasted and 4% overweight. 46% of infants were breastfed within the first hour, 63% were described as exclusively breastfed under 6 months, and breastfeeding continued for 12 months in 74%. The indicator for introduction of solids was met for 41% of infants. Only 13% of children satisfied the indicator for minimum dietary diversity, 43% achieved minimum meal frequency, and 5% had a minimally acceptable diet. About 63% of infants had had sugary snacks in the preceding 24 h, rising to 78% in the second year. Fried and salted snack foods had been eaten by 34% of infants and 66% of children under two. Stunting and wasting remain unacceptably common in informal settlements in Mumbai, and IYCF appears problematic, particularly in terms of dietary diversity. The ubiquity of sugary, fried, and salted snack foods is a serious concern: substantial consumption begins in infancy and exceeds that of all other food groups except grains, roots, and tubers.

## Introduction

Global estimates for 2012 suggested that 51 million children under five were wasted (low weight for height), 162 million were stunted (low height for age), and 99 million were underweight (low weight for age) on the basis of indices lower than two standard deviation (*z*) scores below the median for age and sex (UNICEF, WHO 2013). Childhood malnutrition is a public health priority to which one-third of child deaths can be attributed (Vesel et al. 2010), and long-term malnutrition manifest as stunting increases the risk of infections and reduces cognitive development and economic productivity (Fawzi et al. 1997; Victora et al. 2008; Dewey and Begum 2011).

India's economy is growing and malnutrition levels have fallen (Ghosh and Shah 2004; Kanjilal et al. 2010; Subramanyam et al. 2010), but it is still home to half the world's underweight children (Thakur et al. 2011). Progress has been slow (Kapur et al. 2005; Ramachandran 2007; Thakur et al. 2011; Patel et al. 2012), and stunting (48%) and wasting (20%) remain common (Government of India Ministry of Health and Family Welfare 2007). The 2011 Census of India described an urban population of over 377 million (Office of the Registrar General and Census Commissioner 2012), with the expectation of 590 million by 2030 (McKinsey Global Institute 2010). Of 7935 towns and cities, 468 have at least 100,000 residents, 53 have over one million, and three––Mumbai, Delhi, and Kolkata–-have over 10 million. Informal settlements (slums) are common sources of affordable housing (Planning Commission 2011), and an abiding image of urban poverty (Steering Committee on Urbanization 2011). The Census defines them as residential areas characterized by dilapidation, overcrowding, poor arrangement and building design, narrowness or poor arrangement of streets, and lack of ventilation, light, or sanitation. Across India, about 17% of urban households are in informal settlements, although the proportion varies across states: the most recent estimate for Mumbai is 41% (Chandramouli 2011).

The prevalence of malnutrition in informal settlements is generally higher than in other urban areas (Ghosh and Shah 2004). A study in a Delhi settlement described stunting in 74% of children under three and wasting in 19% (Kapur et al. 2005). The proportions were 63% and 27%, respectively, in a settlement in Lucknow (Awasthi and Pande 1997), and higher than in an earlier study (Saxena et al. 1997). Worrying levels of stunting (35%) and underweight (>40%) were also seen in children under five in a settlement in Pune (Rao et al. 2000). Few studies have documented the nutritional status of children living in Mumbai's informal settlements. The National Family Health Survey suggested that 47% of children were stunted, 16% wasted, and 36% underweight (IIPS and Macro International 2008). We have found similar prevalences (Das et al. 2012).

Undernutrition is––self-evidently, but not exclusively––a product of infant and child feeding practices, within a network of modifiers that include child age, sex, maternal education, socioeconomic status, recurrent infection, low birth weight, and uptake of antenatal care. Indicators describing infant and young child feeding (IYCF) were developed by WHO in 1991 and revised in 2007 (WHO 2008). Table1 summarizes eight core indicators. Adequate IYCF would seem to be crucial for growth and development (Ghosh and Shah 2004; Sinhababu et al. 2010; Kuryan and Kurpad 2012), and may also prevent short- and long-term morbidity (Kuryan and Kurpad 2012). It has been suggested that ∽20% of under-five deaths could be prevented if all IYCF indicators were achieved (Sinhababu et al. 2010), and that ∽22% of neonatal deaths could be averted with optimal breastfeeding (Edmond et al. 2006).

**Table 1:** Core infant and young child feeding (IYCF) indicators recommended by WHO (WHO 2008)

Indicator	Age group	Definition
Early initiation of breastfeeding	0–23 months	Breastfeeding initiated within one hour of birth.
Exclusive breastfeeding under 6 months	0–5 months	Children fed only breastmilk.
Continued breastfeeding at 1 year	12–15 months	Children who continue to be breastfed.
Timely introduction of solid, semi-solid and soft foods	6–8 months	Infants fed solid, semi-solid or soft foods.
Minimum dietary diversity	6–23 months	Infants fed foods from 4 or more food groups.
Minimum meal frequency	6–23 months	Infants fed the minimum number of times or more: 2 times per day for 6–<9 month breastfed infants. 3 times per day for 9–<23 month breastfed children. 4 times per day for 6–<23 months nonbreastfed children.
Minimum acceptable diet	6–23 months	Composite of minimum dietary diversity and minimum meal frequency.
Consumption of iron-rich or iron fortified foods	6–23 months	Children fed iron-rich or iron-fortified food made for infants or fortified in the home.

We used secondary data from a census of families in 40 urban informal settlement areas of Mumbai to address two objectives: to document the prevalences of stunting, wasting, underweight, and overweight in children under five, and to understand the extent to which WHO core IYCF indicators were met.

## Materials and Methods

### Setting

Data were collected over 18 months from September 2011 to March 2013, in a baseline census for a cluster randomized controlled trial of community resource centers in informal settlements (Shah More et al. 2013). The census covered 40 localities selected after a systematic vulnerability assessment in two municipal wards (Osrin et al. 2011). A secondary analysis of these data is presented here.

### Data collection

Two teams of five data collectors and one supervisor demarcated localities of 600 households each. Homes were visited up to three times each to arrange interviews with married women aged 15–49 years. Questionnaires included modules on resident numbers, age, livelihoods, and schooling, assets and utilities, and pregnancies in the preceding 2 years. Information on feeding was collected for children aged 0–24 months, according to WHO guidelines (WHO 2010a). Questions covered types of complementary foods and liquids. Nonmilk fluids included plain water, sugar or glucose water, gripe water, sugar-salt solution, fruit juice, infant formula, tea, honey, and traditional drops. Questionnaires were completed by one team per cluster of homes, after which children under 5 years were gathered for anthropometry. Weight was measured on Seca 385 electronic scales accurate to 10 g. Length was measured with Harlow rollameters accurate to 1 mm. Height was measured with Leicester stadiometers accurate to 1 mm. All measurements were made twice and the mean value was used in the analysis. Relative technical error of measurement (%TEM) was assessed 6-monthly, with representative values of 0.02% for weight measurements and 0.6% for length/height.

### Statistical analysis

We excluded children over 5 years, and twins since their nutritional status is known to differ from that of singletons (Buckler and Green 2004). Wealth was described by asset scores derived from standardized weights for the first component of a principal components analysis (including home ownership, possession of a ration card, robust housing fabric, private water supply, private toilet, finished floor, and possession of a mattress, pressure cooker, gas cylinder, stove, bed, table, clock, mixer, telephone, refrigerator, or television (Filmer and Pritchett 2001; Vyas and Kumaranayake 2006)). Maternal schooling was described by an ordered categorical variable, as none, primary, entered secondary, completed secondary, or higher than secondary. We generated standard deviation (*z*) scores for weight for age, height for age, weight for height, and body mass index for age using the 2006 WHO growth standards and the *zscore06* module in Stata/IC 13.1 (StataCorp, College Station, TX) (zscore06). Outliers were removed such that height for age *z* score ranged from −6 to +6, weight for height from −5 to +5, weight for age from −6 to +5, and body mass index for age from −5 to +5 (WHO Expert Committee on Physical Status 1995). We derived binary variables describing moderate (lower than −2 *z* scores) and severe (lower than −3 *z* scores) stunting, wasting, and underweight. Overweight was defined as a body mass index for age greater than +1 and up to +2 *z* scores, and obesity greater than +2. Eight core binary IYCF indicators were coded on the basis of feeding records as recommended by the WHO (WHO 2008), using definitions summarized in Table1 and allowing for breastfeeding status. The WHO defines snacks as foods eaten between meals, usually self-fed and easy to prepare (WHO 2005). Snacks may be nutritious, but, in accordance with the recommendations of the WHO and World Health Assembly, we are concerned about snack foods that contain high levels of sugar, salt, and fat (WHO 2010b). We asked parents about sugary snacks (chocolates, sweets, candies, pastries, cakes or biscuits) and salted, fried snacks (*nalli*, *wafers*, or crisps). We also asked about two common meals: packet noodles (often referred to as *Maggi*) and *vada pav*, a potato patty in a roll.

We tabulated frequencies and proportions for characteristics of households and mothers and described continuous variables with means and standard deviations (SD). We plotted *z* scores against child age and fitted quadratic regression lines with 95% confidence intervals (CI). We tabulated means and standard deviations of *z* scores, overall, by sex and by age group in years, along with frequencies and percentages of stunting, wasting, underweight, overweight, and obesity. Fulfillment of core WHO IYCF indicators was tabulated in the same way. We examined associations between each of the eight indicators and height for age *z* score (HAZ) or weight for height *z* score (WHZ) in linear models, and stunting or wasting in logit models. We entered HAZ, WHZ, stunting, or wasting as dependent variables in separate models with each of the eight IYCF indicators as an independent variable. We accounted for the clustered nature of the data by using generalized estimating equations with an exchangeable correlation matrix and robust standard errors (Hayes and Moulton 2009). The findings are presented as odds ratios with 95% confidence intervals. We selected covariates for inclusion in adjusted models on the basis of our findings from previous analyses (Das et al. 2012). Adjusted models with HAZ or stunting as the dependent variable included covariates for asset score, parity, maternal age, and maternal education. Models for WHZ or wasting included the same covariates with the addition of child age.

### Ethical statement

The study was approved by the Multi-institutional Ethics Committee, Mumbai, and the University College London Research Ethics Committee.

### Role of the funding source

The sponsors had no role in the study design, data collection, analysis, interpretation, or writing of the article. The corresponding author had access to all study data and responsibility for the decision to submit for publication.

## Results

**Figure 1:**
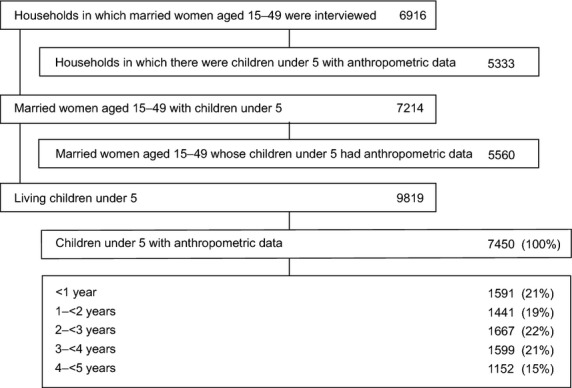
Study flow chart.

Figure1 shows the study flow chart. We measured 7450 children under five born to 5560 women (76% of the children enumerated by their mothers). About one-fifth of children fell under each annual age group, with some drop-off in the fifth year due to absence at preschool. Households had a median six residents and about half of families said that they owned their homes, the majority of which were of robust construction (Table2). Most homes had electricity, but two-thirds of families had to buy water from a tanker or in containers and use of public toilets was the norm. About 60% of mothers said that they had at least begun secondary schooling, but few were engaged in remunerated work. Most families in the area were Muslim and 18% of women had five or more children.

**Table 2:** Characteristics of mothers of children under 5 years in 40 informal settlements of Mumbai, 2011–2013

	*N*	%
Households	5273	100
Home ownership		
Own home	2836	54
Rented home	2437	46
Housing construction		
Robust (pucca)	3010	57
Partly robust (semi-pucca)	1336	25
Temporary (kaccha)	927	18
Electricity supply		
None	10	<1
Metered	3231	61
Family pay landlord	522	10
Other	1510	29
Drinking water source		
Private tap	870	16
Community tapstand	858	16
Buy from tanker or in containers	3545	67
Toilet facility		
Private	529	10
Public	4685	89
None available	59	1
Women	5443	100
Schooling		
None	1769	32
Primary (class 1–5)	373	7
Entered secondary (class 6–9)	2300	42
Completed secondary (class 10)	618	11
Higher than secondary	383	7
Livelihood		
Not earning	5206	96
Unskilled work	137	2
Other	98	2
Missing	2	
Religion		
Muslim	4574	84
Hindu	847	16
Other	22	<1
Parity		
1	1274	23
2	1420	26
3	1099	20
4	692	12
5 or greater	954	18
Missing	4	

Table3 summarizes the anthropometric findings. Mean height for age was −1.68 *z* scores, with little difference between the sexes. About 45% of children under five were stunted, 19% severely so. Wasting was less common: acute malnutrition was identifiable in 16% of children (18% of boys and 15% of girls), of whom 4% were in the severe category. The proportion of stunting increased from 15% in the first year to 56% in the fifth. Moderate or severe acute malnutrition was particularly common in the first year of life (27%), stabilizing at around 13% in the third year. About 4% of children under five were overweight and 1% were obese.

**Table 3:** Standard deviation (*z*) scores for anthropometric indicators, overall and by sex and age group, for singleton children under 5 years in 40 informal settlements in Mumbai, 2011–2013

Index	Children	*z* score		*z* score <−2		*z* score <−3	
	*N*	Mean	SD	*n*	%	*n*	%
Height for age							
All	7356	−1.68	1.68	3290	44.7	1419	19.3
Boys	3781	−1.67	1.69	1693	44.8	721	19.1
Girls	3575	−1.68	1.67	1597	44.7	698	19.5
<1 year	1563	−0.23	1.82	236	15.1	80	5.1
1–<2 years	1424	−1.83	1.53	664	46.6	288	20.2
2–<3 years	1649	−2.08	1.45	877	53.2	397	24.1
3–<4 years	1582	−2.16	1.37	878	55.5	403	25.5
4–<5 years	1138	−2.21	1.19	635	55.8	251	22.1
Weight for height							
All	7381	−0.99	1.14	1209	16.4	325	4.4
Boys	3787	−1.03	1.17	681	18.0	192	5.1
Girls	3594	−0.93	1.10	528	14.7	133	3.7
<1 year	1558	−1.17	1.43	427	27.4	162	10.4
1–<2 years	1427	−0.96	1.18	235	16.5	70	4.9
2–<3 years	1654	−0.88	1.04	202	12.2	41	2.5
3–<4 years	1593	−0.94	0.97	200	12.6	30	1.9
4–<5 year	1149	−0.99	9.95	145	12.6	22	1.9
Weight for age							
All	7430	−1.66	1.23	2884	38.8	942	12.7
Boys	3820	−1.66	1.22	1482	38.8	480	12.6
Girls	3610	−1.66	1.24	1402	38.8	462	12.8
<1 year	1583	−0.99	1.40	331	20.9	111	7.0
1–<2 years	1437	−1.64	1.17	535	37.2	173	12.0
2–<3 years	1663	−1.81	1.11	715	43.0	213	12.8
3–<4 years	1597	−1.94	1.10	736	46.1	261	16.3
4–<5 years	1150	−2.01	1.00	567	49.3	184	16.0

Body mass index for age				*z* score >1		*z* score >2	
All	7391	−0.78	1.16	386	5.2	76	1.0
Boys	3792	−0.80	1.19	206	5.4	40	1.0
Girls	3599	−0.76	1.12	180	5.0	36	1.0
<1 year	1566	−1.17	1.35	80	5.1	20	1.3
1–<2 years	1423	−0.66	1.21	105	7.4	20	1.4
2–<3 years	1657	−0.60	1.10	100	6.0	20	1.2
3–<4 years	1595	−0.69	0.99	65	4.1	8	0.7
4–<5 years	1150	−0.79	0.94	36	3.1	8	0.7

**Figure 2:**
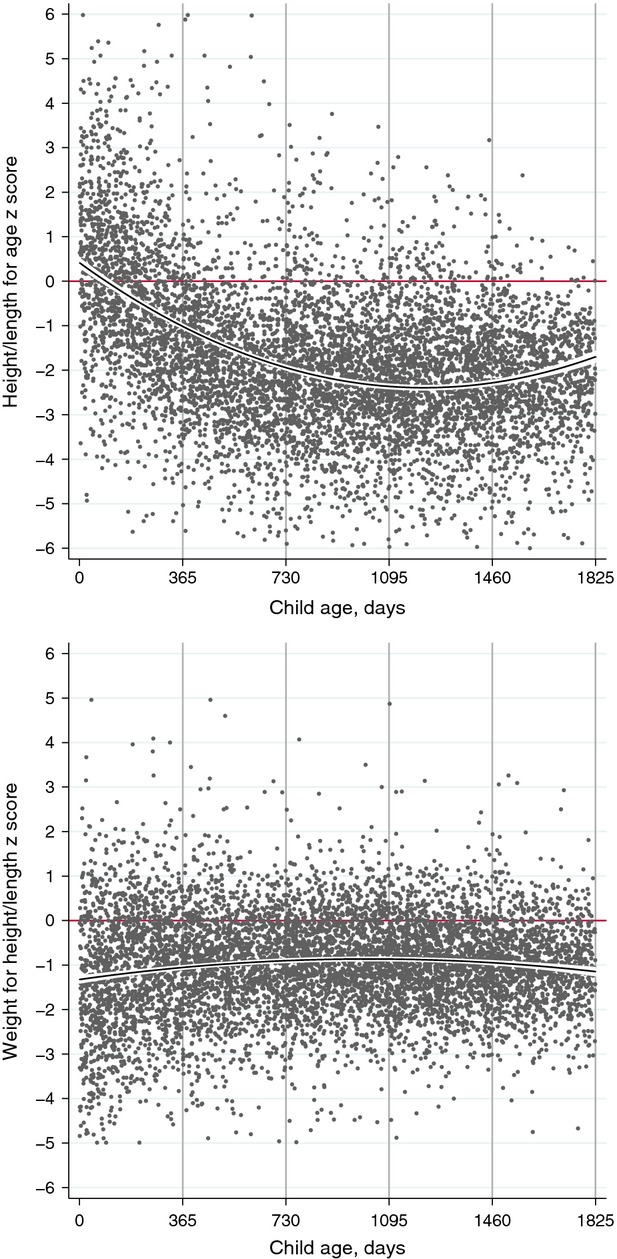
Standard deviation (*z*) scores for anthropometric indicators by age, showing quadratic line of fit with 95% confidence intervals, for singleton children under 5 years in 40 informal settlements of Mumbai, 2011–2013.

These variations in the prevalence of stunting and wasting by age group are illustrated in Figure2, which presents the findings for each child by age. The impression is of a fall in length/height for age from birth to a nadir of ∽−2 *z* scores at around 3 years of age. Weight for height *z* scores increased from birth and stabilized at ∽−1 *z* scores by 1 year of age.

Table4 summarizes the findings for IYCF core indicators. Just under half of infants were reported as being breastfed within the first hour, roughly two-thirds were described as being exclusively breastfed under 6 months of age, and breastfeeding continued to a year in three-quarters of infants. The indicator for introduction of solids was met for 41% of infants, although reported consumption of iron-rich foods was low at 15%. There were no obvious differences between the sexes. Composite indicators were particularly poor: diets lacked diversity––only 13% of children met the criterion––and, when combined with a 43% achievement of suggested meal frequency, only 5% of children had a minimally acceptable diet.

**Table 4:** Fulfillment of core Infant and Young Child Feeding indicators, overall and by sex and age group, for singleton children under 2 years in 40 informal settlements in Mumbai, 2011–2013

IYCF indicator	Children	Children fulfilling indicator	
	*N*	*n*	%
Early initiation of breastfeeding (0–23 month)			
All	3886	1788	46.0
Boys	2032	919	45.2
Girls	1810	834	46.1
0–11 months	2029	926	45.6
12–23 years	1857	862	46.4
Exclusive breastfeeding under 6 months (0–5 month)			
All	1063	666	62.6
Boys	550	341	62.0
Girls	503	319	63.4
Continued breastfeeding at 1 year (12–15 month)			
All	698	513	73.5
Boys	370	279	75.4
Girls	323	231	71.5
Introduction of solids or semisolids (6–8 month)			
All	502	207	41.2
Boys	253	113	44.7
Girls	242	92	38.0
Minimum dietary diversity (6–23 month)			
All	2902	377	13.0
Boys	1522	201	13.2
Girls	1345	175	13.0
6–11 months	999	58	5.8
12–23 months	1903	319	16.8
Minimum meal frequency (6–23 months)			
All	2902	1257	43.3
Boys	1522	653	42.9
Girls	1345	590	43.9
6–11 months	999	332	33.2
12–23 months	1903	925	48.6
Minimum acceptable diet (6–23 month)			
All	2902	137	4.7
Boys	1522	73	4.8
Girls	1345	64	4.8
6–11 months	999	30	3.0
12–23 months	1903	107	5.6
Consumption of iron-rich foods (6–23 month)			
All	2902	439	15.1
Boys	1522	244	16.0
Girls	1345	189	14.1
6–11 months	999	178	17.8
12–23 months	1903	261	13.7

**Figure 3:**
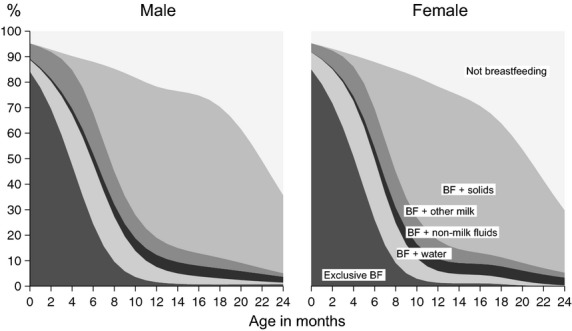
Types of breastfeeding by age, for singleton children under 2 years in 40 informal settlements in Mumbai, 2011–2013.

Figure3 presents more detailed information on breastfeeding. Exclusive breastfeeding, irrespective of prelacteals, began for over 80% of infants, but had largely ended by 6 months. About 20% of infants were given water along with breastfeeds and another 10% with nonbreast milks. Solids were introduced relatively early, although around 20% of infants had not received solids by 12 months of age. There were no obvious differences between the sexes.

Table5 presents univariable and multivariable odds ratios for the association of each IYCF indicator with HAZ, stunting, WHZ, and wasting in children under 24 months. Achievement of none of the indicators was associated with increased HAZ or WHZ, or a reduction in the odds of stunting or wasting. Achievement of the minimum dietary diversity indicator was associated with reduced odds of wasting in unadjusted, but not in adjusted, models.

**Table 5:** Associations between achievement of 8 core IYCF indicators by children aged 0–23 months and anthropometric indicators of malnutrition: height for age, stunting, weight for height, and wasting

IYCF indicator fulfilled	Height for age					*N*	Weight for height					*N*
	HAZ	No stunting	Stunting				WHZ	No wasting	Wasting			
	Coefficient (95% CI)	*n* (%)	*n* (%)	OR (95% CI)	aOR^1^ (95% CI)		Coefficient (95% CI)	*n* (%)	*n* (%)	OR (95% CI)	aOR^2^ (95% CI)	
Early initiation of breastfeeding (0–23 months)	0.03 (−0.01, 0.06)	921 (44.6)	421 (47.4)	1.12 (0.95, 1.31)	1.12 (0.95, 1.31)	2954	0.00 (−0.03, 0.04)	1050 (45.6)	288 (44.2)	0.99 (0.81, 1.20)	1.01 (0.83, 1.23)	2951
Exclusive breastfeeding under 6 months (0–5 months)	0.02 (−0.02, 0.05)	465 (62.8)	49 (66.2)	1.22 (0.76, 1.97)	1.22 (0.77, 1.97)	815	−0.04 (−0.11, 0.02)	341 (62.4)	167 (62.6)	0.95 (0.73, 1.23)	0.82 (0.61, 1.10)	813
Continued breastfeeding at 1 year (12–15 months)	0.06 (−0.03, 0.16)	252 (74.3)	143 (79.9)	1.37 (0.88, 2.12)	1.34 (0.84, 2.15)	518	0.00 (-.08, 0.09)	320 (75.8)	73 (76.8)	1.04 (0.61, 1.78)	1.04 (0.58, 1.84)	517
Introduction of solids or semisolids (6–8 months)	−0.03 (−0.10, 0.04)	137 (44.3)	25 (40.3)	0.84 (0.50, 1.38)	0.83 (0.50, 1.39)	371	−0.01 (−0.09, 0.06)	132 (44.3)	29 (40.3)	0.84 (0.53, 1.35)	0.90 (0.54, 1.51)	370
Minimum dietary diversity (6–23 month)	0.03 (−0.04, 0.09)	174 (13.1)	114 (13.9)	1.10 (0.84, 1.42)	1.12 (0.86, 1.45)	2146	−0.04 (−0.08, 0.01)	247 (14.0)	40 (10.4)	0.68 (0.47, 0.98)	0.77 (0.53, 1.10)	2145
Minimum meal frequency (6–23 months)	0.04 (−0.00, 0.07)	577 (43.5)	382 (46.7)	1.14 (0.97, 1.34)	1.16 (0.98, 1.39)	2146	0.01 (−0.02, 0.04)	787 (44.7)	171 (44.4)	0.99 (0.80, 1.23)	1.06 (0.86, 1.32)	2145
Minimum acceptable diet (6–23 months)	0.03 (−0.08, 0.15)	64 (4.8)	43 (5.3)	1.13 (0.69, 1.85)	1.16 (0.70, 1.92)	2146	0.01 (−0.07, 0.08)	88 (5.0)	19 (4.9)	0.93 (0.56, 1.55)	1.05 (0.62, 1.80)	2145
Consumption of iron-rich foods (6–23 months)	0.01 (−0.05, 0.07)	215 (16.2)	127 (15.5)	0.96 (0.75, 1.25)	1.04 (0.81, 1.35)	2146	0.00 (−0.03, 0.04)	280 (15.9)	60 (15.6)	0.99 (0.79, 1.24)	1.02 (0.81, 1.30)	2145

IYCF, Infant and Young Child Feeding; HAZ, height for age *z* score; WHZ, weight for height *z* score; OR, odds ratio; aOR, adjusted odds ratio; CI, confidence interval.

1 Adjusted for asset score, parity, maternal age, maternal education.

2 Adjusted for asset score, parity, maternal age, maternal education, child age.

Given the poor dietary diversity indicator, Table6 provides a more detailed breakdown of the food groups that infants and children aged 6–23 months were reported to have consumed in the last 24 h. Rice- and wheat-based foods were the commonest, followed by dairy products and legumes. Neither of the latter, however, had been eaten by more than half of the children aged 12–24 months. Less than 15% of children in this age group had eaten flesh foods, eggs, fruit or vegetables. By contrast, over 60% of infants had been given chocolates, sweets, candies or pastries and the figure rose to almost 75% at 12–24 months. Fried and salted snack foods such as potato chips, *chakli*, or samosas had been eaten by one-third of infants and two-thirds of children under 2 years. The association of snack consumption with wealth quintile was unclear. Nonparametric tests for trend suggested that consumption of sugary and savory snacks was lower in wealthier groups, but this was not supported by logistic regression analysis that included wealth quintile groups as indicator variables.

**Table 6:** Food groups and snack foods consumed in last 24 h, overall and by sex and age group, for singleton children aged 6–23 months in 40 informal settlements in Mumbai, 2011–2013

Food group	Children	Consumed	
	*N*	*n*	%
Grains, roots or tubers			
All	2822	1799	63.8
Boys	1472	930	63.2
Girls	1316	851	64.7
6–11 months	999	406	40.6
12–23 months	1823	1393	76.4
Legumes or nuts			
All	2822	1144	40.5
Boys	1472	588	40.0
Girls	1316	547	41.6
6–11 months	999	263	26.3
12–23 months	1823	881	48.3
Dairy: milk, yogurt or cheese			
All	2822	1267	44.9
Boys	1472	655	44.5
Girls	1316	601	45.7
6–11 months	999	382	38.2
12–23 months	1823	885	48.6
Flesh foods: meat, fish, poultry, liver, organ meats			
All	2822	162	5.7
Boys	1472	87	5.9
Girls	1316	74	5.6
6–11 months	999	15	1.5
12–23 months	1823	147	8.1
Eggs			
All	2822	349	12.4
Boys	1472	138	9.4
Girls	1316	121	9.2
6–11 months	999	46	4.6
12–23 months	1823	215	11.8
Vitamin A rich fruits and vegetables			
All	2822	261	9.2
Boys	1472	138	9.4
Girls	1316	121	9.2
6–11 months	999	46	4.6
12–23 months	1823	215	11.8
Other fruit and vegetables			
All	2822	277	9.8
Boys	1472	141	9.6
Girls	1316	133	10.1
6–11 months	999	46	4.6
12–23 months	1823	231	12.7

Snack foods	Children	Consumed	
	*N*	*n*	%
Sugary foods: chocolates, sweets, candies, pastries			
All	2021	1496	74.0
Boys	1054	768	72.9
Girls	943	709	75.2
6–11 months	553	346	62.6
12–23 months	1468	1150	78.3
Wealth quintile 1 (poorest)	435	323	74.2
Wealth quintile 2	372	291	78.2
Wealth quintile 3	395	295	74.7
Wealth quintile 4	382	295	77.2
Wealth quintile 5 (least poor)	437	292	66.8
Salted, fried snacks (*wafers*, crisps, *nalli*)			
All	2019	1156	57.3
Boys	1054	590	56.0
Girls	941	552	58.7
6–11 months	553	187	33.8
12–23 months	1466	969	66.1
Wealth quintile 1 (poorest)	434	261	60.1
Wealth quintile 2	372	247	66.4
Wealth quintile 3	395	245	62.0
Wealth quintile 4	382	192	50.3
Wealth quintile 5 (least poor)	436	211	48.4
Packet noodles or potato burger (*vada pav*)			
All	2020	127	6.3
Boys	1053	61	5.8
Girls	943	65	6.9
6–11 months	553	14	2.5
12–23 months	1467	113	7.7
Sweetened soft drinks			
All	2820	187	6.6
Boys	1469	98	6.7
Girls	1318	89	6.8
6–11 months	1042	22	2.1
12–23 months	1778	165	9.3

## Discussion

In an analysis including over 7000 children less than 5 years in 40 Mumbai informal settlements, we identified stunting in 45% and wasting in 16%. The prevalence of stunting appeared to increase over the first 2 years of life, while wasting reduced over the early months. IYCF indicators in children under two were poor, particularly in terms of dietary diversity and acceptable diet. The strengths of the study were its focus on children living in Mumbai's poorer informal settlements, its large sample size, and the quality of the anthropometric data. Its main weakness––a weakness general to the literature––was its cross-sectional nature, which makes causal inference difficult.

### Comparison of anthropometry with other studies

India's third National Family Health Survey (NFHS-3) included slum populations in Mumbai and can be used as an anthropometric and dietary comparator. Height and weight were measured for children under 5 years and the methods of dietary data collection were similar to the ones we used, albeit with fewer questions. The NFHS-3 consisted of three questionnaires, of which the Woman's Questionnaire (administered to women aged 15–49 who were usual residents of the sample household, or visitors who had stayed the night before the survey) provided data on infant and young child feeding (Government of India Ministry of Health and Family Welfare 2006). Questions covered breastfeeding status (ever breastfed, early initiation, and breastfeeding continuation, with median duration for youngest-born children within 3 years preceding the survey). Information on supplementary foods was obtained through 24 h recall (Government of India Ministry of Health and Family Welfare 2007).

The prevalences of stunting and wasting were similar to the 47% and 16% found by the Mumbai informal settlement analysis of the NFHS-3. Severe acute malnutrition prevalence was also similar (4%) (IIPS and Macro International 2008). In comparison with prevalences elsewhere, stunting was common, but levels of wasting were similar. For example, national data from Bangladesh described stunting in 34% and wasting in 21% of children (Zongrone et al. 2012). Growth restriction begins before birth (Mamidi et al. 2011; Das et al. 2012), and other studies have found the burden of stunting and wasting largest in children under two (Kumar et al. 2006; Victora et al. 2010; Mathad et al. 2011), but this is not certain: older children were at greater risk of stunting in Karnataka and Uttar Pradesh (Brennan et al. 2004), and moderate and severe malnutrition were greatest between 2 and 3 years of age in urban informal settlements in Delhi (Kapur et al. 2005).

### Comparison of IYCF with other studies

There were no apparent sex differences in the odds of meeting any of the IYCF indicators. The figures for early initiation of breastfeeding were higher than reported by the NFHS-3, but exclusive breastfeeding under 6 months followed the pattern of the literature, which suggests that breastfeeding in India is almost universal, but that only half of infants under 6 months are exclusively breastfed and a quarter are put to the breast within the first hour of life (Government of India Ministry of Health and Family Welfare 2007). The early initiation rate of 46% was slightly lower than the NFHS-3 figure of 50% for children living in Mumbai informal settlements (IIPS and Macro International 2008). It was also lower than the figure found in informal settlements in Nairobi, Kenya, where 63% of infants were put to the breast in the first hour of life. Exclusive breastfeeding was more common: only 2% of infants in Nairobi slums under 6 months were exclusively breastfed and two in five were given something to drink other than breastmilk within 3 days of birth (Kimani-Murage et al. 2011).

Early and exclusive breastfeeding is important (Edmond et al. 2007; Meshram et al. 2012). In a study in Delhi, mortality risk was seven times greater for diarrhea and five times greater for pneumonia in children who were not exclusively breastfed (Khan et al. 2012). Our figure of 46% for early initiation accords with the literature. Rates of early initiation are low both nationally (Ghosh and Shah 2004; Government of India Ministry of Health and Family Welfare 2007; Ramji 2009), and in local studies (Bhosale et al. 1997; IIPS and Macro International 2008; Oommen et al. 2009; Meshram et al. 2012). Less than half of mothers report exclusive breastfeeding (Ghosh and Shah 2004; Government of India Ministry of Health and Family Welfare 2007; Ramachandran 2007; Ramji 2009), particularly in informal settlements (Kumar et al. 1992; Aneja et al. 2001; Sethi et al. 2003; Ghosh and Shah 2004; Roy et al. 2009). The NFHS-3 recorded a mean duration of breastfeeding of 2.7 months in Mumbai and a proportion of 53% across Maharashtra (IIPS and Macro International 2008). Our figure of 63% is higher than the state average. We recorded a rate of breastfeeding continuation of 74% at 1 year, similar to an earlier study from Mumbai informal settlements (78%) (Bavdekar et al. 1994). The NFHS-3 figure was 89% (Patel et al. 2010), and studies in urban and rural India have found continuation rates of over 85% (Bhosale et al. 1997; Garg and Chadha 2009; Sinhababu et al. 2010).

The indicators for introduction of solids and minimum meal frequency were met by less than half of infants, similar to figures reported by other studies (Government of India Ministry of Health and Family Welfare 2007; Ramachandran 2007; Garg and Chadha 2009; Ramji 2009; Patel et al. 2010; Kuryan and Kurpad 2012; Senarath and Dibley 2012). The NFHS-3 figure for Mumbai informal settlements was 35% in children under two (IIPS and Macro International 2008). Weaning probably tends to be later in urban informal settlements, with an average age of introduction of 10 months (Ghosh and Shah 2004), and only 17% of infants achieving the indicator in some areas in Delhi (Kuryan and Kurpad 2012). Few studies have looked at Mumbai settlements, but one found a rate of 48% in 1994 (Bavdekar et al. 1994). In addition to the infants at age 6 months who did not meet the timely introduction of solid or semi-solid foods indicator, at age 12 months over 20% of children had still not been fed solid food, which would support the idea of late weaning. Cost is one of several potential explanations for this. The minimum meal frequency indicator was met for fewer than half the infants in our study. The all-India proportion for achievement of the recommended minimum meal frequency is 40–45% (Government of India Ministry of Health and Family Welfare 2007; Patel et al. 2010; Senarath and Dibley 2012), the NFHS-3 figure for Mumbai informal settlements being 50% in children under two (IIPS and Macro International 2008). Rural populations fare better (61% at 6–8 months of age) (Garg and Chadha 2009).

Dietary diversity was particularly limited (13%) and only 5% of young children had a minimum acceptable diet. Once solids are introduced, their range is limited. The NFHS-3 suggested that 32% of children in Mumbai informal settlements achieved the minimum dietary diversity indicator (much more than we found) (IIPS and Macro International 2008), but other studies have found rates of ∽15–30% (Garg and Chadha 2009; Patel et al. 2010; Khan et al. 2012; Senarath and Dibley 2012), and figures ranging from ∽40% to 70% have been found across other Asian countries such as Bangladesh, Nepal, and Sri Lanka (Senarath and Dibley 2012). A Delhi study found that consumption of almost every food group apart from milk was inadequate and that children were only receiving 56% of their recommended daily energy intake (Kapur et al. 2005). A study in the same area put the figure at 75% (Aneja et al. 2001). Assessments of minimum acceptable diet–– a composite of dietary diversity and meal frequency––are scarce. Available data suggest that only 9% of children across India meet the indicator (Patel et al. 2010; Senarath and Dibley 2012). In Bangladesh, Nepal, and Sri Lanka, a minimum acceptable diet was achieved by ∽40–68% of infants (Senarath and Dibley 2012), although these studies used national datasets and are not directly comparable with ours. Minimum dietary diversity and minimum acceptable diet were met by <10% of infants using national data from Ethiopia, which is more in line with our findings (Disha et al. 2012). Consumption of iron-rich or iron-fortified foods is similarly hard to judge, although the NFHS-3 suggested that 70% of children under five might be anemic (Government of India Ministry of Health and Family Welfare 2007).

Our findings fit into a mixed and complex landscape of infant and young child feeding in low- and middle-income countries. Variation across Asia is clear, with timely introduction of complementary foods reaching around 70% in Bangladesh and Nepal, as high as 84% in Sri Lanka, but only 39% in Pakistan (Senarath and Dibley 2012). Variation is also apparent further afield, with the same indicator at 90% in Zambia, but only 61% in Ethiopia (Disha et al. 2012). In informal settlements in Nairobi, 98% of children had been introduced to complementary foods by 6 months (Kimani-Murage et al. 2011).

### Lack of association between IYCF and anthropometric indicators

The WHO IYCF indicators represent consensual best feeding practice and a potential means of tracking progress epidemiologically. The lack of association between achievement of either breastfeeding or complementary feeding indicators and HAZ or WHZ accords with the literature. Although an analysis of pooled DHS data from 14 low-income countries suggested that meeting all the core IYCF indicators except minimum meal frequency was associated with less likelihood of stunting or underweight (Marriott et al. 2012), the situation is less clear. A recent review examined relationships between core IYCF indicators and HAZ, WHZ, stunting, and wasting from studies based on DHS data. Most of the analyses included child age, child sex, and an asset-based wealth measure in models, and all but one included maternal education (Jones et al. 2014).

Early initiation of breastfeeding was positively associated with HAZ in 1 of 8 datasets, exclusive breastfeeding under 6 months was negatively associated with HAZ in seven datasets (two significantly), and continued breastfeeding at 12 months was negatively associated with HAZ in two datasets, with supporting trends in others. Timely introduction of solids was positively associated with HAZ in two datasets (and negatively associated with stunting in 1), the minimum dietary diversity indicator was positively associated with HAZ in three datasets, including India (where it was negatively associated with stunting: OR 0.76; 95% CI 0.65, 0.89). Minimum acceptable diet was positively associated with HAZ in three datasets, including India, but neither minimum meal frequency nor the iron-rich food indicator was associated with HAZ or stunting in any dataset. There was no association of early initiation of breastfeeding, continued breastfeeding at 12 months, minimum dietary diversity, or iron-rich foods with WHZ or wasting. Exclusive breastfeeding and minimum meal frequency were positively associated with WHZ (and negatively associated with wasting) in one dataset. Counterintuitively, timely introduction of solids was negatively associated with WHZ at 6–8 months, and minimum acceptable diet was positively associated with wasting in one dataset (Jones et al. 2014).

There are a number of possible explanations for the lack of clear association between the achievement of IYCF indicators and anthropometric outcomes. The indicators themselves may not be sensitive or specific enough in terms of dietary quality at an individual level. Misclassification is likely to be common for breastfeeding indicators (reporting of exclusive breastfeeding is, in our experience, particularly questionable). There is some evidence that early initiation of breastfeeding prevents stunting (Brennan et al. 2004; Kumar et al. 2006; Ramji 2009), but some studies have found no association with breastfeeding practices (Kumar et al. 2006; Vesel et al. 2010; Zongrone et al. 2012; Menon et al. 2013). Nevertheless, studies in Karnataka, Uttar Pradesh, Maharashtra, and across India have suggested that exclusive breastfeeding protects against malnutrition (Rao et al. 2000; Brennan et al. 2004; Ramji 2009). In addition to breastfeeding practices, some studies suggest that appropriate complementary feeding benefits nutritional outcomes (Sinhababu et al. 2010; Mamidi et al. 2011; Disha et al. 2012). In a study in rural Uttar Pradesh, a composite complementary feeding index was associated with improvement in stunting, but not in wasting (Garg and Chadha 2009). Delayed introduction of solids has been associated with both stunting and underweight in some settings (Disha et al. 2012; Patel et al. 2012; Zongrone et al. 2012; Menon et al. 2013). That dietary diversity is beneficial for child nutrition has been documented in a number of countries, including India (Arimond and Ruel 2004; Garg and Chadha 2009; Disha et al. 2012; Zongrone et al. 2012; Menon et al. 2013).

Reverse causality may also play a part. Mothers and families might be concerned about infants who are not doing as well as others––particularly if they are wasted–and make efforts to ensure that they are fed as well as possible (Jones et al. 2014). We think that it would be a coincidence if these efforts neutralized any associations so comprehensively, although the cell sizes we achieved might have been too small to show associations because indicators were assessed for tight age bands. For example, the denominator for continued breastfeeding at 1 year included 685 children and this was reflected in broad confidence intervals.

Finally, some indicators are likely to be more important than others and the uniformly poor achievement of a minimum acceptable diet may have compromised all the children in our sample. Or perhaps the burden of exposure to intestinal pathogens and other infections overrides the effect of feeding. It is also possible that the effects of IYCF are not manifest as contemporaneous somatic growth, or may not manifest until children are older. Some of the indicators are assessed over narrow windows and it may be unreasonable to look for short-term benefit. For example, introduction of solids is assessed for children at 6–9 months of age and its effects on growth may not appear until later.

### Snack foods

There are growing concerns about the double burden of stunting and overweight (Black et al. 2013), and informal settlements in Mumbai might be an indicator of the situation in low-income urban settings. Of particular concern is the combination of nutrient-poor diet and unhealthy snacks. Huffman et al. (2014) have recently used Demographic and Health Survey data to examine the consumption of processed snack foods by children in 18 low- and middle-income countries. Although the analysis did not include India, sugary snacks were eaten by 34–68% of children aged 6–23 months in Asian datasets. Consumption increased with age and wealth quintile, and urban children were more likely than rural children to have eaten sugary snacks, which were themselves more likely to have been eaten than eggs and vitamin A-rich fruits. Data from African datasets suggested that consumption of sugary snack food was slightly lower than in Asia, at ∽18–40%.

Our study confirms these findings–-outstrips them, in fact––and adds some detail. In the same age group, sugary snacks were consumed by 74% of children and savory snacks by 57%. Substantial consumption began in infancy and there was no obvious association with sex or wealth (bearing in mind that the highest quintile group were the least poor inhabitants of informal settlements, rather than the urban wealthy). If anything, it may have been that the poorest infants and children received more snack foods. Perhaps the low unit cost of a snack makes it an easier option within a busy day. Given the low levels of overweight and obesity in this young group of children, the primary current concern may not be the double burden of undernutrition and overweight, but the contribution of limited diversity and replacement of nutritious foods with snacks to endemic stunting. Early feeding practices may set children's later food preferences to a degree (Ventura and Mennella 2011; Stein et al. 2012). We can only speculate on the physiological consequences (Niinikoski and Ruottinen 2012), but the combination of empty calories, sugar, and salt is a serious concern.

## Conclusion

High proportions of stunting and wasting represent a nutrition crisis in Mumbai's informal settlements and we welcome the shift of focus to infants and younger children that is currently being made by India's Integrated Child Development Services. An obvious next step is to follow a cohort of infants in order to understand individual trajectories and temporal challenges to growth. We are currently doing this through monthly visits to infants from birth to 1 year of age, in order to develop a longitudinal impression of growth and its associations with serial reports of feeding and illness, as well as with a range of other exposures.
